# Micelle-Assisted Synthesis of Al_2_O_3_ *·*CaO Nanocatalyst: Optical Properties and Their Applications in Photodegradation of 2,4,6-Trinitrophenol

**DOI:** 10.1155/2013/641420

**Published:** 2013-11-07

**Authors:** Ayesha Imtiaz, Muhammad Akhyar Farrukh, Muhammad Khaleeq-ur-rahman, Rohana Adnan

**Affiliations:** ^1^Department of Chemistry, GC University Lahore, 54000 Lahore, Pakistan; ^2^School of Chemical Sciences, Universiti Sains Malaysia, 11800 Pulau Pinang, Malaysia

## Abstract

Calcium oxide (CaO) nanoparticles are known to exhibit unique property due to their high adsorption capacity and good catalytic activity. In this work the CaO nanocatalysts were prepared by hydrothermal method using anionic surfactant, sodium dodecyl sulphate (SDS), as a templating agent. The as-synthesized nanocatalysts were further used as substrate for the synthesis of alumina doped calcium oxide (Al_2_O_3_ *·*CaO) nanocatalysts via deposition-precipitation method at the isoelectric point of CaO. The Al_2_O_3_ *·*CaO nanocatalysts were characterized by FTIR, XRD, TGA, TEM, and FESEM techniques. The catalytic efficiencies of these nanocatalysts were studied for the photodegradation of 2,4,6-trinitrophenol (2,4,6-TNP), which is an industrial pollutant, spectrophotometrically. The effect of surfactant and temperature on size of nanocatalysts was also studied. The smallest particle size and highest percentage of degradation were observed at critical micelle concentration of the surfactant. The direct optical band gap of the Al_2_O_3_ *·*CaO nanocatalyst was found as 3.3 eV.

## 1. Introduction

Metal oxide nanoparticles play effective role in degradation of hazardous chemicals. Their highly intrinsic surface area and catalytic properties have made them destructive adsorbents. These metal oxides not only adsorb hazardous chemicals on their surface but also destroy them into smaller and less harmful by-products [1, 2]. They can destroy a wide range of such chemicals, for example, chlorobenzenes, organosulfurs, organophosphates, and nitroaromatics. These chemicals are present or used in synthesis of explosive materials, insecticides, pollutants, and chemical warfare agents [3–15]. Their increased consumption and improper disposal have become serious environmental risk. Some of them are penetrating into the ground water through soil, causing serious environmental and health problems [16].

Nitrophenols (NPs) are primarily concerned toxic pollutants by the United States Environmental Protection Agency (USEPA) [17], as these compounds have been found in industrial and agricultural wastes. These are anthropogenic, noxious, inhibitory, and biorefractory organic compounds and are considered as hazardous substances [18]. Among these, 2,4-dinitrophenol (2,4-DNP), 2,5-dinitrophenol (2,5-DNP), 2,6-trinitrophenol (2,6-DNP), and 2,4,6-TNP (2,4,6-TNP) are the most common and multipurpose industrial chemicals with wide-ranging applications as insecticides, dyes, drugs, and ordnance compounds [19–25]. Due to biorefractory properties of these pollutants, the biological techniques appear futile or take protracted incubation time on the degradation [26–28]. Thus, it is significantly important to develop new remediation methods for the decomposition of these organic pollutants.

Magnesium oxide nanoparticles were found as an effective adsorbent for the 2,4-dinitrotoluene (2,4-DNT) and 2,4,6-trinitrotoluene (2,4,6-TNT) [29]. Photocatalytic degradation of nitrophenols and nitroamines was also observed on titanium oxide [12, 30, 31], iron oxide [16], and gold loaded aluminium oxide [32]. Activity of calcium oxide nanoparticles was studied against the degradation of dimethyl methylphosphonate (DMMP) and it was found that these nanoparticles have the ability to degrade other warfare chemical agents [5, 33]. Degradation of such compounds was also observed when aluminium oxide nanoparticles were used [3, 6, 9, 32].

Surfactants play an important role in the preparation of metal oxide nanoparticles because of their influence on particle growth, coagulation, and flocculation. Under hydrothermal condition, smallest particle size was reported by using anionic surfactant (SDS) as compared to cationic surfactant (CTABr) and nonionic surfactant (PEG) [34, 35]. Several methods like anodization [36], wet oxidation [37], sol-gel [38], hydrothermal treatment [34], deposition precipitation [22, 32], and thermal vapor deposition [39] methods are being applied for the synthesis of nanoparticles. 

In the current study, CaO and Al_2_O_3_·CaO nanocatalysts were synthesized using hydrothermal method by varying the concentration of sodium dodecyl sulphate (SDS), an anionic surfactant. The objective of this study is to assess the photodegradation of selected ordnance compound by CaO and Al_2_O_3_·CaO nanocatalysts. The ordnance compound of concern, 2,4,6-TNP (2,4,6-trinitrophenol or picric acid), was considered of priority for this study. The effects of temperature and surfactant were studied for the synthesis of nanocatalysts, and catalytic properties of nanocatalysts were assessed for degradation of 2,4,6-TNP. 

## 2. Materials and Methods

### 2.1. Materials

Calcium chloride anhydrous (CaCl_2_), sodium hydroxide (NaOH), and sodium dodecyl sulfate (SDS) were purchased from Merck while aluminium chloride (AlCl_3_·6H_2_O) and methanol (CH_3_OH) were from Riedel-de Haën. For the catalytic reaction, 2,4,6-TNP was purchased from Riedel-de Haën. All chemicals were used as received, without any further purification.

### 2.2. Characterization

The CaO nanocatalysts obtained were subjected to thermogravimetric analysis (TGA) by using SDT Q600 TGA. Structural analysis of CaO and Al_2_O_3_·CaO nanocatalysts was done using Fourier Transform Infrared (FTIR)—MIDAC 2000 with KBr powder and powder X-ray diffractometer (XRD) using PANalytical MPD X'PERT PRO. The diffraction patterns were compared using the standard database from International Centre for Diffraction Data (ICDD). The morphology and particle size of nanocatalysts was determined by FEI quanta 200 F Field Emission Scanning Electron Microscope (FESEM) and Philips CM12, 80 kV, Transmission Electron Microscope (TEM). HPLC analysis was performed on Shimadzu model LC 20-AT instrument equipped with diode array detector (SPD-M20A, Shimadzu). Chromatographic separation was performed by using C-18 column (250 × 4.6 mm, 5 *μ*m packing) with isocratic solution. An injection volume of 20 *μ*L was used for each sample. The peaks were observed at wavelength of 355 nm. GC-MS analyses were performed on Shimadzu model QP-2010 instrument. 

### 2.3. Synthesis of CaO Nanocatalysts by Hydrothermal Method

**Figure 1:**
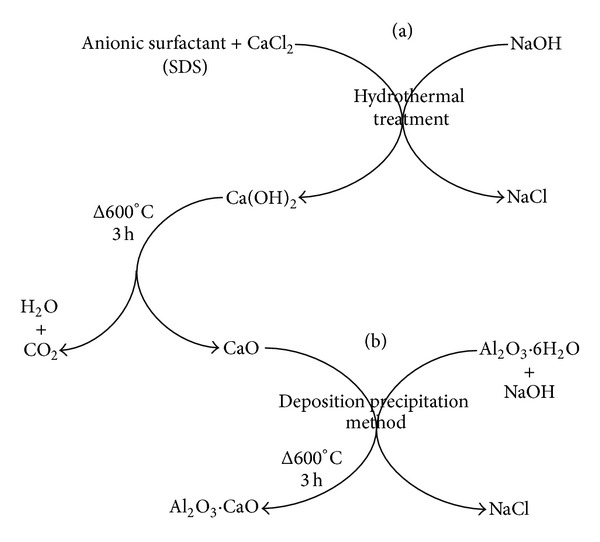
Experimental scheme for the synthesis of (a) CaO and (b) Al_2_O_3_·CaO nanocatalysts.

CaO nanocatalysts were synthesized by changing the experimental parameters, that is, temperature and concentration of surfactant, to study their effect on particle size and catalytic activity. Synthesis method of CaO is depicted in Figure 1(a).

The mixture containing 0.15 M of CaCl_2_ and 0.008 M sodium dodecyl sulfate (SDS) was magnetically stirred at ambient temperature. The precursor to surfactant molar ratio was taken as 1 M : 0.05 M. Sodium hydroxide (0.30 M) solution was added dropwise and the reaction solution was stirred for 30 minutes. After stirring, the reaction suspension was placed in a Teflon line autoclave (hydrothermal bomb) and kept in an oven for 4 h at the desired temperatures (250, 180, 160, and 140°C).

After 4 h, the autoclave was removed from the oven and allowed to cool for 2 h at ambient temperature. The precipitates of Ca(OH)_2_ were separated and washed 3 times with methanol and 2 times with deionized water to remove any reactant, ions, or surfactant and neutralize their pH by using centrifugation machine at the speed of 13000 rpm. The precipitates were dried and calcined at 600°C in a furnace with air flow for 3 h [40]. 

Similar process (Figure 1(a)) was adopted to study the effect of surfactant on CaO nanocatalysts. Only the molar concentration of SDS (0.004, 0.006, 0.008, 0.01, and 0.012 M) was varied to be close—and far from its critical micelle concentration (CMC) value which is 8.1 mM [41]. 

### 2.4. Preparation of Alumina Supported CaO Nanocatalysts by Deposition Precipitation Method

Synthesis of alumina doped CaO nanocatalysts was carried out by deposition precipitation method [32]. AlCl_3_·6H_2_O and CaO nanocatalysts were used as precursors. 15 mM solution of AlCl_3_·6H_2_O was prepared in 9 mL deionised water and 50 mg of CaO nanocatalysts was added at different time intervals to attain pH 12.3 (isoelectric point) [42]. Meanwhile, the reaction was constantly stirred on magnetic stirring plate. Teflon line autoclave was filled with reaction solution and kept in the oven for 4 h at the same temperature as that of CaO nanocatalysts precursor. Similarly, the precipitates were washed and calcined. The experimental setup is displayed in Figure 1(b).

## 3. Results and Discussion

### 3.1. Thermogravimetric Analyses

**Figure 2:**
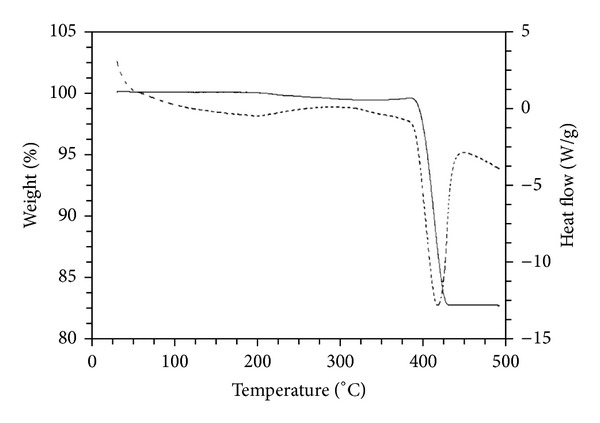
TGA (solid line)/DSC (dotted line) plots of uncalcined sample prepared with 0.008 M SDS at 180°C for 4 h.

Figure 2 shows TGA/DSC profile of the Ca(OH)_2_ synthesized with SDS at 180°C for 4 h via hydrothermal treatment. A significant weight loss (16.25%) is observed in the temperature range 375–450°C, which can be attributed to the thermal decomposition of Ca(OH)_2_. The observed weight loss in the range is smaller than the theoretical value (24.3%) calculated on the assumption of total dehydration of Ca(OH)_2_ to CaO [40].

The results indicate that a nearly complete conversion of Ca(OH)_2_ to CaO took place below 600°C. This implies that the surfactant used in the fabrication of CaO nanocatalysts had been almost removed at around 450°C.

### 3.2. Fourier Transform Infrared Analyses

**Figure 3:**
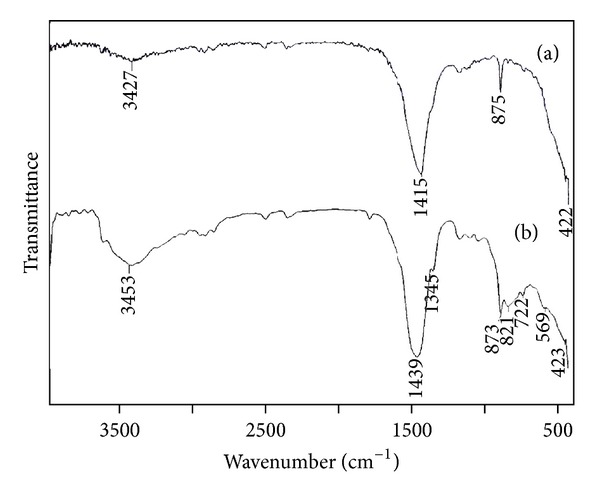
FTIR spectra for (a) CaO fabricated with surfactant (SDS) and (b) Al_2_O_3_·CaO nanocatalysts.

FTIR peaks (Figure 3) at 3427 cm^−1^ and 3453 cm^−1^ can be attributed to the stretching and bending vibrations of hydrogen-bonded surface OH groups (physisorbed water). It reveals that only a slight amount of water molecules is retained in the fabricated CaO and Al_2_O_3_·CaO samples. The appearance of strong IR absorption band at 424 cm^−1^ may be attributed to the lattice vibrations of CaO [43]. The IR absorption bands at 1415 cm^−1^ and 1439 cm^−1^ are due to the symmetric stretching vibration of unidentate carbonate. Weak absorption band at 875 cm^−1^ further demonstrates the presence of carbonate species. This is due to exposure of highly reactive surface area of CaO to air during calcination which resulted in the formation of considerable amount of CO_2_ and H_2_O, which are adsorbed on the surface of CaO in the form of free –OH and carbonate species. This indicates that surface –OH and lattice oxygen of CaO do provide oxygen which is more assessable on high surface area samples (Figure 3(a)) [44, 45]. In Figure 3(b), the peaks at 821 cm^−1^, 722 cm^−1^, and 569 cm^−1^ are due to the stretching vibration of Al–O bond [46].

### 3.3. X-Ray Diffraction Analyses

**Figure 4:**
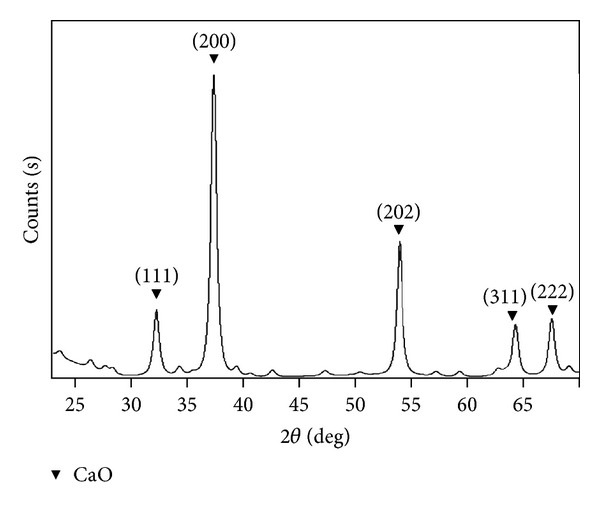
XRD pattern of CaO nanocatalyst fabricated with surfactant.

The XRD pattern (Figure 4) of the CaO obtained with SDS heated at 180°C for 4 h after hydrothermal treatment shows that the nanocatalysts can be indexed to cubic CaO. Their lattice parameters agree well with the corresponding standard values given in JCPDS PDF# 82-1690 (CaO). The intense peaks at 32.2°, 37.3°, 54.5°, 64.5°, and 67.3° correspond to the (111), (200), (202), (311), and (222) crystal planes, respectively. The average crystallite size (3.15 nm) was determined from the broadenings of corresponding peaks by using Scherrer's equation:

(1) $$\begin{array}{c} D=\frac{k\lambda }{\beta \left(\cos\theta \right)}, \end{array}$$

where *D* is the mean crystallite size, *k* is the grain shape dependent constant 0.89, *λ* is the wavelength of the incident beam in nm, *θ* is the Bragg reflection angle, and *β* is the line broadening at half the maximum intensity in radians.

**Figure 5:**
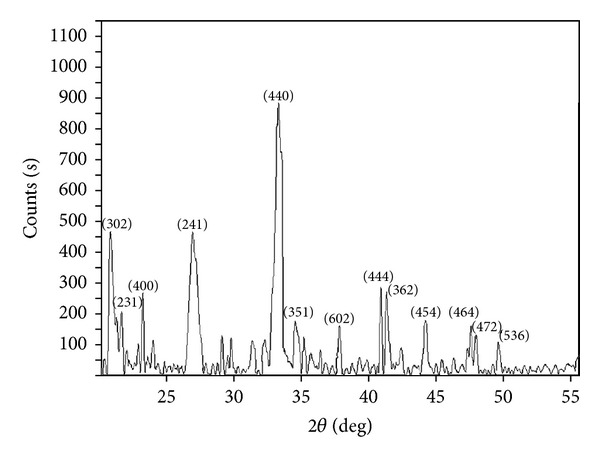
XRD pattern of Al_2_O_3_·CaO nanocatalyst.

The 2*θ* values of Al_2_O_3_·CaO nanocatalysts (Figure 5) were compared with the ICDD database to identify the phase purity and composition formed. Aluminium forms Ca_3_Al_2_O_6_ phase (PDF# 00-006-0495,) with the calcium oxide nanoparticles at 600°C due to strong Al–O interaction [47, 48]. The essential peaks at 2*θ* = 20.9°, 21.8°, 23.3°, 26.7°, 33.2°, 34.8°, 37.2°, 40.8°, 41.4°, 44.8°, 48.8°, 49.6°, and 49.9° correspond to the lattice planes (302), (231), (400), (241), (440), (351), (602), (444), (362), (454), (464), (472), and (536), respectively. The average crystallite size of 3.64 nm for Ca_3_Al_2_O_6 _ was calculated by using (1). Uniform incorporation and distribution of aluminium into the CaO matrix may be responsible for the smaller crystallite size [49].

### 3.4. Optical Properties of Al_2_O_3_·CaO Nanocatalysts

**Figure 6:**
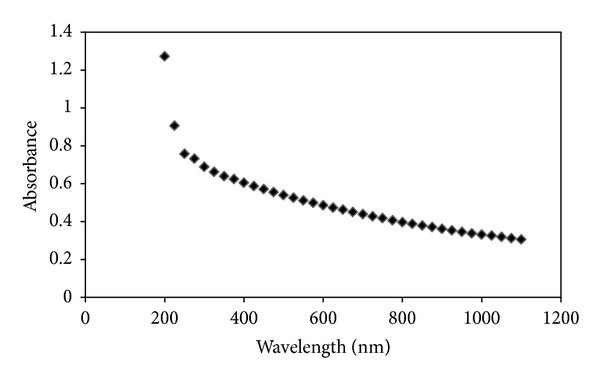
UV-Vis spectra for Al_2_O_3_·CaO nanocatalyst.

Optical properties of Al_2_O_3_·CaO nanocatalysts were analyzed by UV-Vis absorption measurement at room temperature and using deionised water as blank. The sample was prepared by dispersing 3.6 mg of Al_2_O_3_·CaO nanocatalysts in 10 mL of deionised water and stirring by magnetic stirrer for 15 min. A homogeneous suspension solution was prepared and subjected for assessment for optical properties.  Figure 6 describes typical absorption spectra of Al_2_O_3_·CaO nanocatalysts, which shows the shifting of absorption edges to the shorter wavelength (blue shift).

Equation (2) was used to calculate optical absorption coefficient *α* from absorption data:

(2) $$\begin{array}{c} \alpha =2.303\frac{10^{3}\rho A}{lcM}, \end{array}$$

where *ρ* is the theoretical density of Al_2_O_3_·CaO (3.03 g cm^−3^), *A* is the absorbance of Al_2_O_3_·CaO nanocatalyst solution, *l* is the optical path length of quartz cell (1 cm), *c* is the molar concentration of suspension solution, and *M* is the molecular weight of Al_2_O_3_·CaO nanocatalysts. 

**Figure 7:**
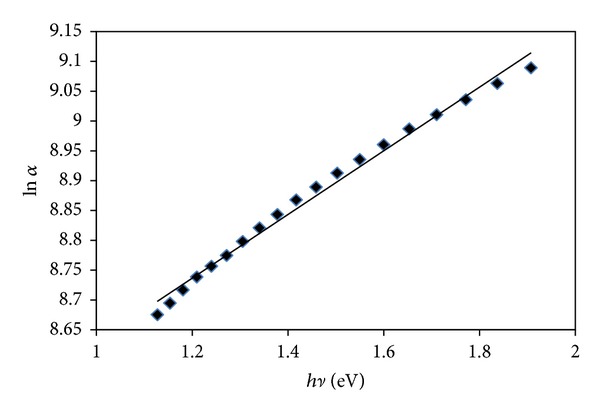
ln⁡*α* versus *hν* for determination of the localized tail state *E*_*e*_.

Using Urbach's equation (3), the density of the localized tail state (*E*_*e*_ = 1.87 eV) in the forbidden energy gap was determined by plotting ln⁡*α* versus *hν* as shown in Figure 7:

(3) $$\begin{array}{c} \alpha =\alpha_{0}e^{h\nu /E_{e}}. \end{array}$$

Here, *α*_0_ a is constant and *hν* is the energy of photons.

**Figure 8:**
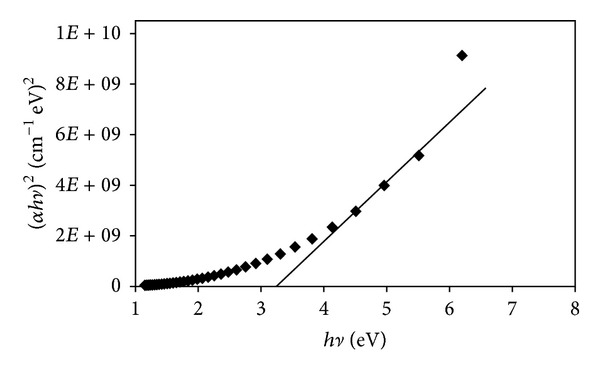
Plot of (*α* *hν*)^2^ versus *hν* of Al_2_O_3_·CaO nanocatalysts.

The optical band gap for direct transition was determined by plotting (*α* *hν*)^2^ versus *hν* using

(4) $$\begin{array}{c} \alpha h\nu =B{\left(h\nu -E_{g}\right)}^{n}, \end{array}$$

where *B* is constant and nature of transition *n* has been assumed to have values 1/2, 2, 3/2, and 3 for direct, indirect, forbidden direct, and forbidden indirect transitions, respectively [50, 51]. The direct optical band gap energy *E*_*g*_ is determined by extrapolating the linear portion of the curve in Figure 8; the intersection of the extrapolation gives the value of 3.3 eV, which is much less than band gap energy of Al_2_O_3_ (7.2 eV) [52]. 

**Figure 9:**
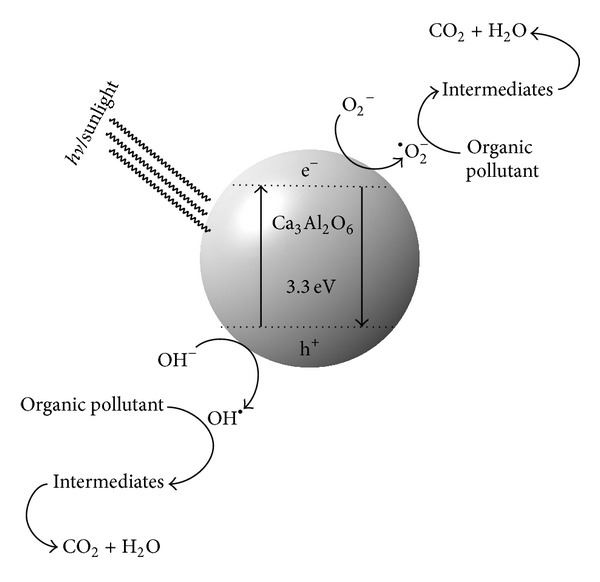
Mechanism of action of Al_2_O_3_·CaO nanocatalyst.

Proposed behavior of Al_2_O_3_·CaO nanocatalyst towards organic pollutant due to band gap energy is illustrated in Figure 9. 

### 3.5. FESEM of CaO and Al_2_O_3_·CaO Nanocatalysts

**Figure 10:**
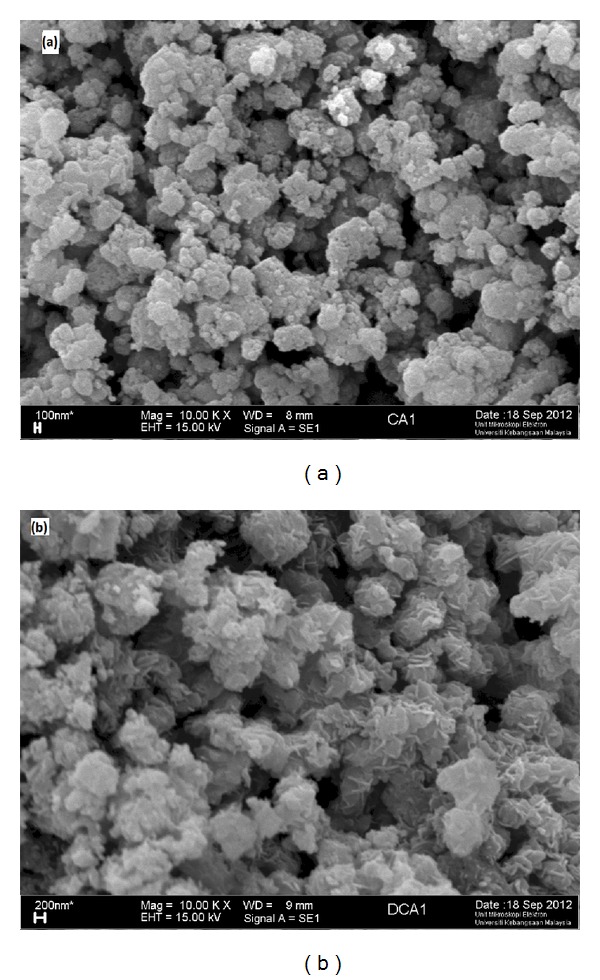
FESEM images for (a) CaO nanocatalysts fabricated with surfactant and (b) alumina supported CaO nanocatalysts.

Figures 10(a) and 10(b) provide the representative FESEM images of the CaO and Al_2_O_3_·CaO nanocatalysts fabricated with surfactant-assisted hydrothermal treatment at 180°C for 4 h and calcination at 600°C for 3 h. It was observed in Figure 10(a) that the CaO samples contain rounded coagulated nanocatalysts. After doping of alumina on CaO nanocatalysts, agglomerated particles of sample were observed in Figure 10(b).

### 3.6. TEM of CaO and Al_2_O_3_·CaO Nanocatalysts 

**Figure 11:**
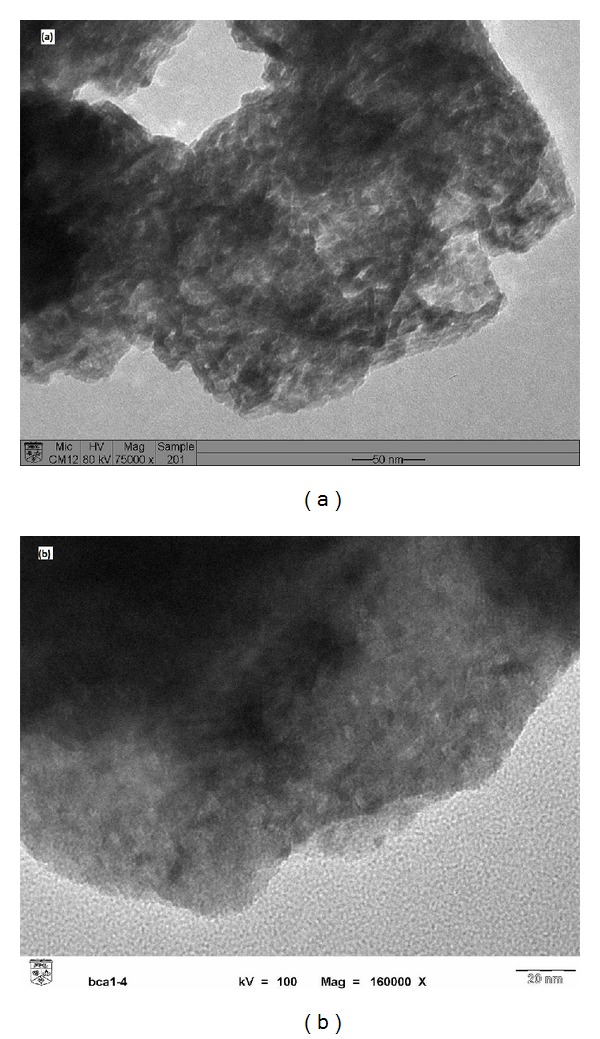
TEM images of (a) CaO and (b) Al_2_O_3_·CaO nanocatalysts fabricated with surfactant (SDS).

Representative TEM image of the CaO and Al_2_O_3_·CaO nanocatalysts obtained after hydrothermal treatment is shown in Figure 11. The nanocatalysts exist in coagulated form with the particle size of 16 nm (Figure 11(a)). The particles size is decreased to 3.6 nm after the formation of Al_2_O_3_·CaO nanocatalysts (Figure 11(b)).

### 3.7. Catalytic Activity of CaO and Al_2_O_3_·CaO Nanocatalysts

**Figure 12:**
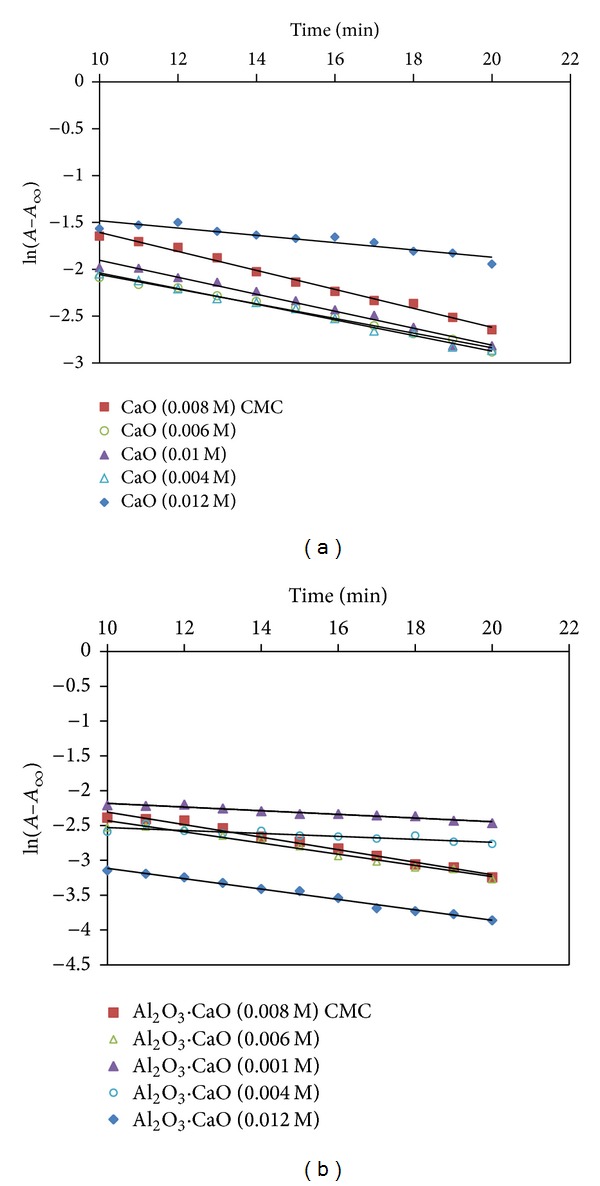
Plot of ln⁡(*A*–*A*_*∞*_) versus time for the oxidation of 2,4,6-TNP with (a) CaO and (b) Al_2_O_3_·CaO nanocatalysts prepared under different concentrations of surfactant.

A mixture of 5 mg of CaO nanocatalysts and 25 mL solution of 2,4,6-TNP (15 ppm) was placed under UV irradiation with constant stirring for 15 minutes at ambient temperature. On the basis of Beer-Lambert law, calibration was done for 2,4,6-TNP at a wavelength of maximum absorptivity, *λ*_max⁡_, 356 nm [53]. The catalytic activity was determined using UV Spectrophotometer (UV-1700 Shimadzu) by measuring the change in absorbance at 356 nm every 60-second interval. Same procedure was adopted to determine catalytic activity of Al_2_O_3_·CaO nanocatalysts against 2,4,6-TNP [54]. The analysis of samples showed a continuous decrease in absorption at *λ*_max⁡_ = 356 nm, which was used to track the degradation of 2,4,6-TNP [55]. It is evident that reaction kinetics of both CaO (Figure 12(a)) and Al_2_O_3_·CaO (Figure 12(b)) nanocatalysts with 2,4,6-TNP follows first order. The first order rate constant values, *k*′, were determined from the slope of the graphs as shown in Figures 12(a) and 12(b). 

### 3.8. Effect of Variation of Temperature on Catalytic Activity of CaO Nanocatalysts

Catalytic activity of CaO nanocatalysts synthesized by varying hydrothermal treatment temperature (140, 160, 180, and 250°C) was studied while keeping other experimental parameters constant. It was observed that an increase in temperature (from 140°C to 180°C) resulted in increases in rate constant, *k* value (0.0732, 0.0791, and 0.1283 min^−1^), but the catalytic activity decreases to 0.1124 min^−1^ at 250°C. This change in the catalytic activity trend suggests that high hydrothermal temperature favors fast reaction which increases the particle size and decreases the surface area and contributes to destructive adsorbent ability.

### 3.9. Effect of Variation of Surfactant Concentration on Catalytic Activity of CaO and Al_2_O_3_·CaO Nanocatalysts

**Figure 13:**
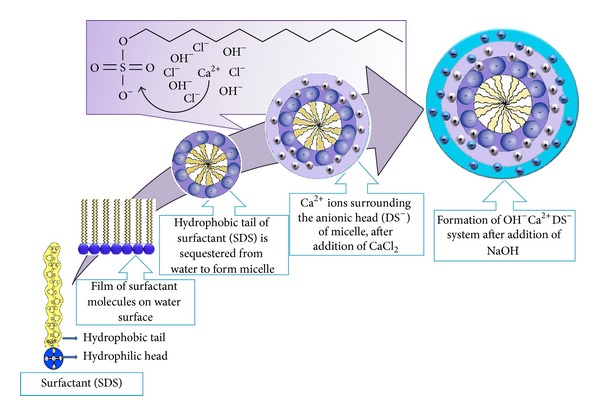
Mechanism of micelle assisted formation of OH^−^Ca^2+^DS^−^ system.

The synthesis of CaO nanocatalysts under basic conditions is believed to follow the X^−^I^+^S^−^ module, where S^−^ is the anionic surfactant, I^+^ is the inorganic precursor, and X^−^ is the counter ion [56]. A generalized mechanism of electrostatic interaction between inorganic precursor, surfactant and counter ions was proposed in Figure 13. When sodium hydroxide is added to the system, Na^+^ and OH^−^ ions are supposed to surround Ca^2+^–DS^−^. The electrostatic attraction between Ca^2+^ and DS^−^ is stronger than that between Na and SD^−^ ions; this behavior enhances the particle formation [57]. Na^+^ joins with Cl^−^ to make NaCl in the mixture system due to the electrostatic repulsion of Cl^−^ and DS^−^. The OH^−^ ions self-assembled around the micelle, so Ca^2+^ ions were attracted towards OH^−^ to form Ca(OH)_2_ in the presence of surfactant (templating agent). In the final step of the process, the template was removed by calcination at 600°C for 3 h to generate pores.

The catalytic activity of CaO and Al_2_O_3_·CaO nanocatalysts (synthesized via hydrothermal treatment at 180°C and 4 h using different surfactant (SDS)) concentration is shown in Tables 1 and 2. It was observed that Al_2_O_3_·CaO nanocatalysts are more effective catalyst for the degradation of 2,4,6-TNP as compared to the CaO nanocatalysts. The rate constant values, *k*, of different samples of CaO and Al_2_O_3_·CaO nanocatalyst at the same parameters were compared. Al_2_O_3_·CaO nanocatalysts have higher rate constant (0.1251 min^−1^) than CaO nanocatalyst (0.1233 min^−1^) at 0.004 M concentration of SDS.

**Figure 14:**
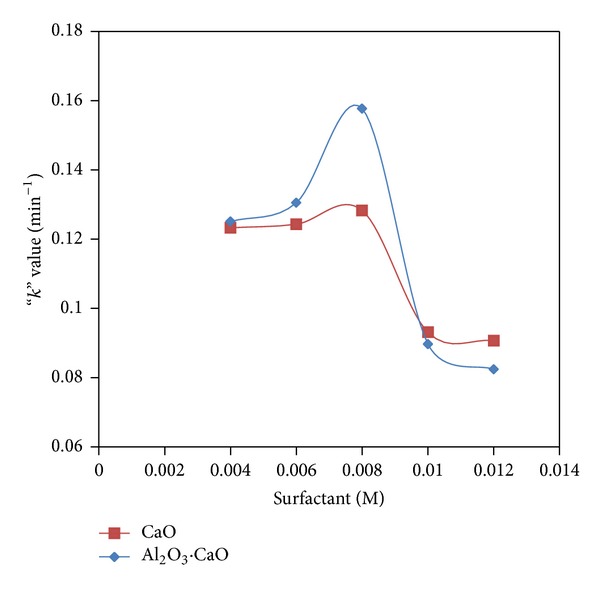
Plot of surfactant concentration versus rate constant “*k*” of the degradation of 2,4,6-TNP.

It was observed that the highest *k* values for both CaO and Al_2_O_3_·CaO were found at CMC of SDS in accordance to the small particle size of these nanocatalysts at this concentration. The *k* value increases (particle size decreases) when the nanocatalysts were prepared by using surfactant from 0.004 to 0.008 M as the precursors are well dispersed in the surfactant template. However, further increase in surfactant concentration from 0.008 to 0.012 M decreases the *k* values and increases the particle size due to formation of micelle which coagulates the particles. A parabola is formed showing the relationship between *k* values and surfactant concentration as shown in Figure 14.

### 3.10. Degradation Mechanism of 2,4,6-TNP

Degradation of 2,4,6-TNP by nanocatalysts was observed by HPLC and GC-MS analysis. A 15 ppm solution of picric acid (0.15 mg) was freshly prepared in 100 mL deionized water and used as standard solution. Mixtures of each (5 mg) calcium oxide and Al_2_O_3_·CaO nanocatalysts were prepared in 25 mL solution of 2,4,6-TNP (15 ppm) and placed under UV irradiation with constant stirring for 15 minutes at ambient temperature. The sample solutions were filtered and then degassed by sonication before use.

**Figure 15:**
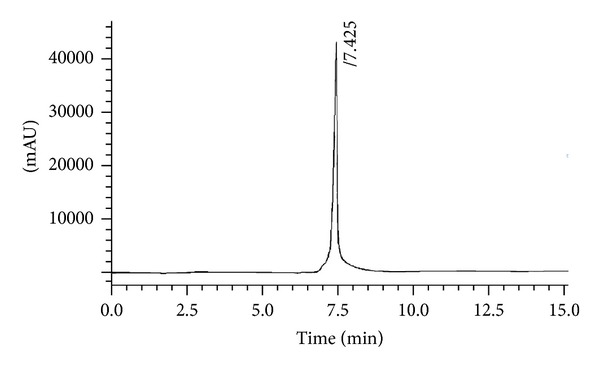
HPLC chromatograph for 2,4,6-TNP.

Mobile phase was prepared for HPLC analyses by mixing 70% methanol with 0.1 M acetic acid buffer in the ratio of 97 : 3, v/v. The mobile phase was filtered and then degassed by sonication before use [58]. The data was analyzed by obtaining area under sample peaks at 355 nm. The observed retention time for standard 2,4,6-TNP solution was found 7.425 min as shown in Figure 15. 

**Figure 16:**
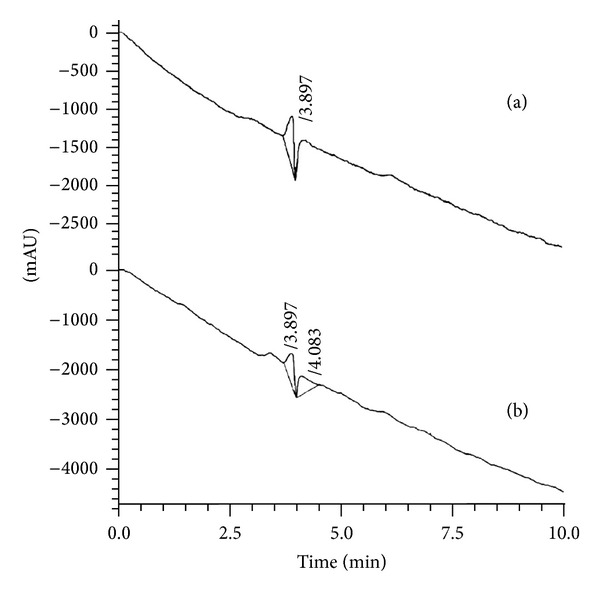
HPLC chromatograph showing the degradation of 2,4,6-TNP.

Each sample solution was injected separately in the HPLC and none of them showed any peak at the wavelength of 355 nm. These results lead to the conclusion that the picric acid was completely degraded by CaO and Al_2_O_3_·CaO nanocatalysts (Figure 16). 

GC-MS technique was used to determine the intermediates generated during catalytic degradation of 2,4,6-TNP. Sample was prepared by suspending 5 mg Al_2_O_3_·CaO nanocatalysts in 25 mL solution of 2,4,6-TNP (15 ppm). Then, it was placed under UV irradiation with constant stirring for 15 minutes at ambient temperature. The sample solution was filtered before use. 

**Figure 17:**
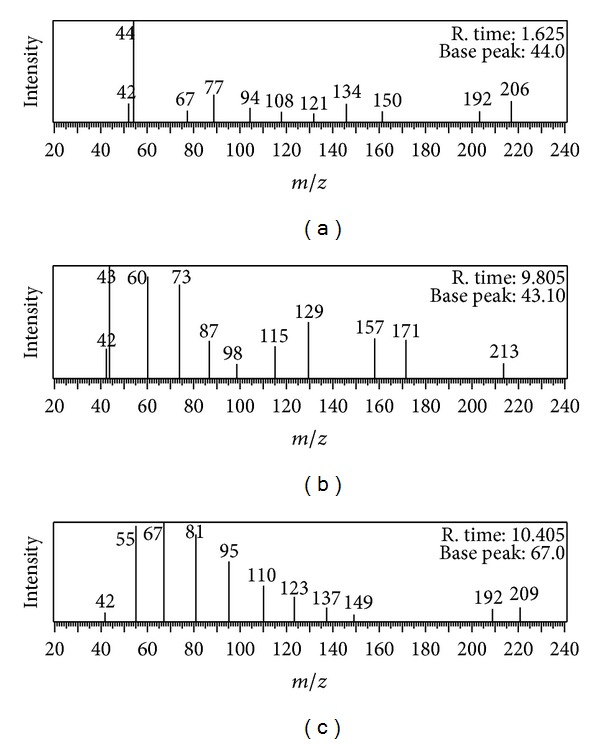
GC-MS chromatograms for degradation of 2,4,6-TNP by Al_2_O_3_·CaO nanocatalyst at retention time (a) 1.65 min, (b) 9.805 min, and (c) 10.405 min.

**Scheme 1:**
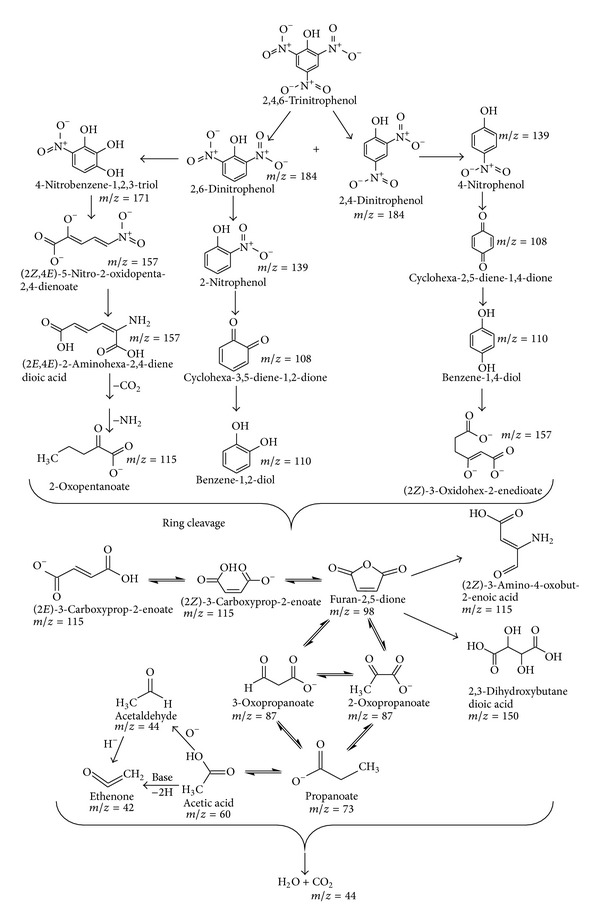
Mechanism for degradation of 2,4,6-TNP by Al_2_O_3_·CaO nanocatalyst.

A schematic diagram is proposed as given in Scheme 1 on the basis of GC-MS chromatogram (Figure 17). 

## 4. Conclusion

CaO and Al_2_O_3_·CaO nanocatalysts were prepared by varying the temperature and surfactant (SDS) concentration above and below CMC value using hydrothermal and using deposition precipitation method. Catalytic activity of these nanocatalysts was measured against the degradation of 2,4,6-TNP, which proved that the nanocatalysts are effective catalysts. The highest rate constant value, *k*, was observed in those samples which were prepared at CMC value of the anionic surfactant. Compared to CaO nanocatalysts, the Al_2_O_3_·CaO nanocatalysts have the highest catalytic activity (0.1577 min^−1^). The band gap of the Al_2_O_3_·CaO nanocatalyst was calculated as 3.3 eV.

## Figures and Tables

**Table 1:** Effect of surfactant concentration on catalytic activity of CaO nanocatalysts prepared at 180°C hydrothermal condition.

As-synthesized sample	Surfactant conc. (M)	“*k*” value (min^−1^)
CaO	0.004	0.1233
CaO	0.006	0.1243
CaO	0.008	0.1283
CaO	0.01	0.0931
CaO	0.012	0.0907

**Table 2:** Effect of surfactant concentration on catalytic activity of Al_2_O_3_·CaO nanocatalysts prepared at 180°C under hydrothermal condition.

As-synthesized sample	Surfactant conc. (M)	“*k*” value (min^−1^)
Al_2_O_3_·CaO	0.004	0.1251
Al_2_O_3_·CaO	0.006	0.1305
Al_2_O_3_·CaO	0.008	0.1577
Al_2_O_3_·CaO	0.01	0.0897
Al_2_O_3_·CaO	0.012	0.0824
